# Insect bite-like reaction in a patient with T-cell lymphoma^☆^

**DOI:** 10.1016/j.abd.2020.12.021

**Published:** 2022-09-08

**Authors:** Tatsuhiko Mori, Kinuko Irie, Toshiyuki Yamamoto

**Affiliations:** Department of Dermatology, Fukushima Medical University, Fukushima, Japan

Dear Editor,

Insect Bite-Like Reaction (IBLR) is a rare skin disorder, which is associated with hematologic malignant neoplasms such as leukemia and malignant lymphoma.^1^ Hematologic malignant neoplasms are derived from B cells in most cases.^2^ We herein describe a rare case of IBLR in Anaplastic Large Cell Lymphoma (ALCL) patient, a type of T-cell lymphoma. To our knowledge, this is the third report of IBLR associated with T-cell lymphoma.

An 84-year-old male was diagnosed with ALK-negative ALCL and treated with chemotherapy (a combination of pirarubicin, cyclophosphamide, vincristine, and prednisolone) in a hospital, which produced complete remission. Two years later, he visited a clinic complaining of pruritic nodules on his hands and back. Biopsy taken from a nodule on his hand revealed prominent lymphocytes in the epidermis, around blood vessels and sweat glands in the dermis as well as diffuse eosinophil infiltration (Fig. 1), and he was diagnosed with Prurigo Nodularis (PN).

**Figure 1:**
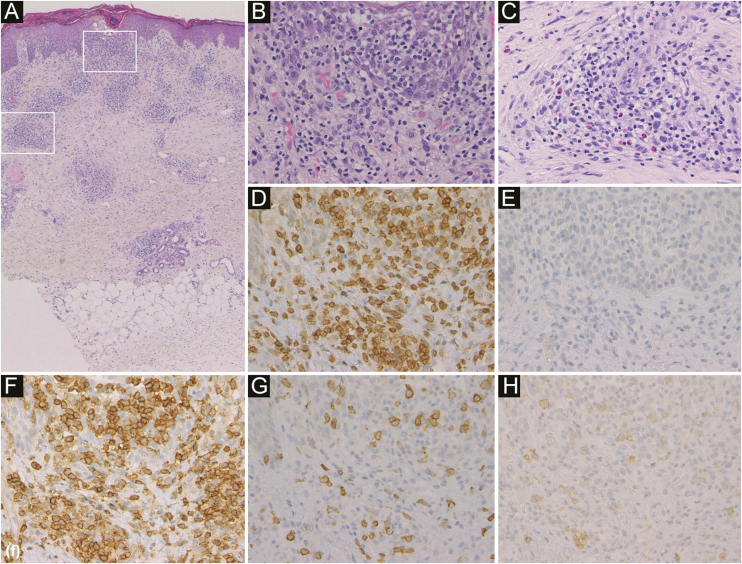
Histological features showed infiltration of inflammatory cells mainly in the epidermis and around blood vessels and sweat glands in the dermis (a). In the epidermis, inflammatory cells were lymphocytes (b). Around blood vessels in the dermis, inflammatory cells were lymphocytes and eosinophils (c) (Hematoxylin & eosin, a ×50; b ×400; c ×400). Immunohistochemistry revealed intense expression of CD3 (d) and CD4 (f), and little expression of CD20 (e), CD8 (g) and CD30 (h) in the infiltrating lymphocytes in the epidermis (original magnification: d–h; ×400).

He visited the same clinic again complaining of nodules with mild pruritus on both cheeks four months after the first visit (Fig. 2). Physical examination showed red or skin color circular nodules of 10 mm in diameter. Laboratory tests showed anemia (hemoglobin 10.0 g/dL), normal levels of eosinophils (688 µL), and slightly elevated levels of lactate dehydrogenase (289 IU/L) and soluble interleukin-2 receptor (750 U/mL). There was no data about serum IgE antibodies. ALCL did not recur at that time. A biopsy from one of the nodules revealed prominent infiltration of lymphocytes, which were mostly CD4 positive non-neoplastic T cells, and eosinophils mainly around blood vessels and sweat glands in the dermis and subcutaneous tissue (Fig. 3). The possibility of a recurrence of ALCL was excluded because CD30 positive neoplastic T-cells were not revealed. He was diagnosed with IBLR. Topical corticosteroid for one month led to the improvement of the skin lesions. Epstein-Barr virus-encoded small RNA in situ hybridization was not detected in either specimen of PN or IBLR.

**Figure 2:**
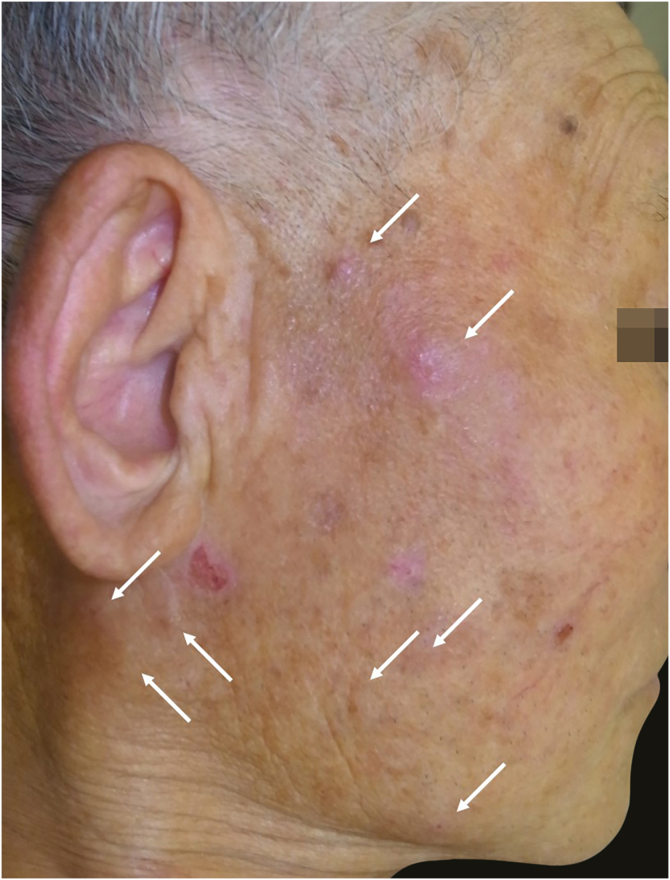
Red or skin color nodules with mild pruritus on the right cheek (arrow).

**Figure 3:**
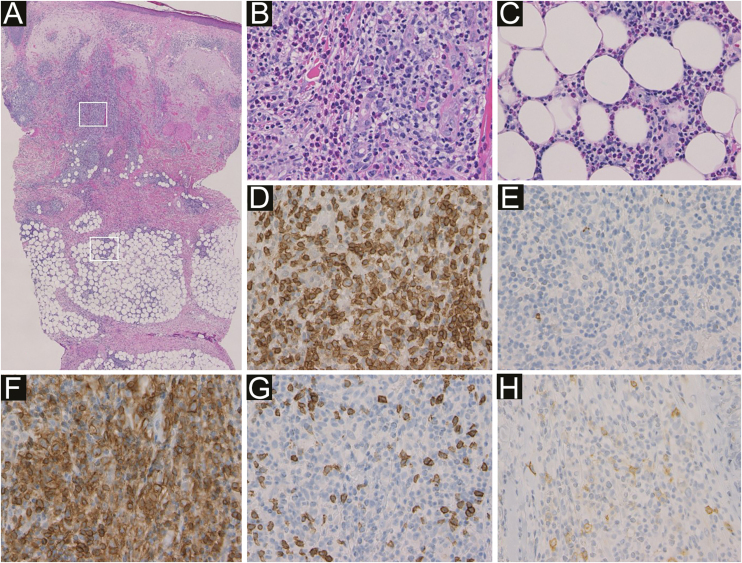
Histological features showed prominent infiltration of inflammatory cells mainly around blood vessels and sweat glands in the dermis and subcutaneous tissue (a). Higher magnification in the dermis (b) and in the subcutaneous tissue (c) showed that inflammatory cells were non-neoplastic lymphocytes and eosinophils (Hematoxylin & eosin, a ×50; b ×400; c ×400). Immunohistochemistry revealed intense expression of CD3 (d) and CD4 (f), and little expression of CD20 (e), CD8 (g) and CD30 (h) in the infiltrating lymphocytes (original magnification: d–h; ×400).

Skin eruptions are common in patients with hematologic malignant neoplasms. Barzilai et al first reported a case of IBLR in which the patient presented with pruritic red papules, nodules, and plaques without insect bite.^1^ IBLR is also called “eosinophilic eruption of hematoproliferative disease”, “exaggerated insect bite reaction”, or “hypersensitivity to insect bite”. Most cases of IBLR are associated with hematologic malignant neoplasms derived from B cells, especially Chronic Lymphocytic Leukemia (CLL). Bairey et al. reported 48 IBLR patients with CLL.^3^ According to their report, IBLR usually appeared after the diagnosis of CLL, while in 29% of the patients, IBLR appeared before the CLL diagnosis. The mean duration of IBLR was 21.5 months. The common sites were lower limbs (79%), upper limbs (67%), and head and neck (38%). Skin eruption of IBLR is clinically similar to that of PN, but PN rarely occurs on the head and neck. Histopathologically, many lymphocytes and eosinophils infiltrate the upper and lower dermis and subcutaneous tissue of the IBLR lesional skin, whereas these inflammatory cells mainly infiltrate the epidermis and upper dermis of the PN lesional skin.^1^ IBLR is a refractory skin disease and is treated with topical or oral corticosteroid, antihistamine, dapsone, and phototherapy, which however are little effective.^1^

The exact pathogenesis of IBLR is still unknown. IBLR has been defined as a disease with nonspecific cutaneous eruption, in contrast to leukemia cutis, which is associated with specific cutaneous lesions. Bairey et al. reported that IBLR was not related to the activity of CLL.^3^ Similar eruptions have been reported in patients with human immunodeficiency virus infection and congenital agammaglobulinaemia.^4^ These past reports indicate that an altered immune reactivity probably plays a causative role in IBLR.^4^ Infiltration of many eosinophils suggested a shift in the Th1/Th2 balance toward Th2. Simon et al. reported that abnormal T-cell clones stimulate the production of eosinophilopoietic cytokines including IL-4 and IL-5, which suggested that in our case IBLR was associated with ALCL.^5^

In our case, PN presented on the patient’s hands and back four months before the appearance of IBLR on his face. ALCL did not recur when these skin diseases appeared. Topical corticosteroids led to the rapid improvement of the IBLR eruption, though IBLR is typically a refractory disease. Biopsy specimens from the back (PN) and the cheek (IBLR) showed infiltration of many eosinophils and CD4 positive non-neoplastic T-cells (Figure 1, Figure 3). These histopathological findings suggest that our patient’s immune balance was shifted toward Th2, which was probably associated with the pathogenesis of PN and IBLR in the present case. To date, 206 IBLR cases have been reported, and only two cases were associated with hematologic malignant neoplasms of T-cell origin.^2^ Therefore, our case is the third report of IBLR associated with T-cell lymphoma. However, it is not yet clear why most IBLR cases are associated with hematologic malignant neoplasms derived from B cells. The pathogenesis of IBLR should be further examined in future studies.

## Financial support

None declared.

## Authors’ contributions

Tatsuhiko Mori: Designed the study; performed the research and contributed to the analysis and interpretation of data; wrote the initial draft of the manuscript; read and approved the final version of the manuscript.

Kinuko Irie: Performed the research and contributed to analysis and interpretation of data; read and approved the final version of the manuscript.

Toshiyuki Yamamoto: Designed the study; assisted in the preparation of the manuscript; read and approved the final version of the manuscript.

## Conflicts of interest

None declared.
